# Mandatory Research Biopsy Requirements Delay Initiation of Clinical Trials

**DOI:** 10.3389/fonc.2019.00968

**Published:** 2019-10-18

**Authors:** Jonathan H. Cheng, Justin W. Tiulim, Sheng Zhou, Anthony El-Khoueiry, Jorge Nieva

**Affiliations:** ^1^LAC+USC Medical Center, Los Angeles, CA, United States; ^2^Department of Medicine, USC Norris Comprehensive Cancer Center, Los Angeles, CA, United States

**Keywords:** biomarker, clinical trial, targeted therapy, immunotherapy, mandatory research biopsy, oncology, lung cancer, head and neck cancer

## Abstract

**Background:** There has been an increasing requirement for fresh tumor tissue to enroll in clinical trials in order to look for specific biomarkers. This has been shown to increase screening duration and increase screen failure rates. It was important to corroborate these results in other centers.

**Methods:** This study is a non-randomized retrospective analysis of patients in one subset of patients seen by research nurses who operated in the standard head/neck and lung team not including patients in the phase 1 program. All patients were enrolled in clinical trials from January 16, 2013 to May 28, 2018 at USC Norris Comprehensive Cancer Institute in Los Angeles. Patients who were required to give fresh research biopsies prior to intervention were part of the research biopsy group.

**Results:** In total, 76 patients were analyzed in this study. Thirty-three patients were in the research biopsygroup and 43 patients were in the no biopsy group. Trials that required a fresh biopsy had a longer median screening duration (30 vs. 14 days) than trials that did not require a biopsy (*p* < 0.0001).

**Conclusions:** Our study shows that requiring biopsies prior to clinical trial treatment results in a statistically significant delay in treatment. The informed consent forms that were part of clinical trials involving mandatory research biopsies did not reflect this delay in treatment. However, these delays did not result in a statistically significant decrease in number of days on trial or days until progression of disease.

## Introduction

The number of biomarker directed clinical trials and cancer treatments is increasing (1). This trend is driven by a series of clinical experiences that demonstrate significant improvement in clinical outcomes and progression free survival in many cancers when biomarker directed therapy is employed (2, 3). Because of these positive results, trial sponsors have increasingly mandated clinical trial designs that require biomarker testing (4). In the United States, the annual spending on clinical trials that involve targeted therapies now exceeds spending on conventional chemotherapies; and there is a resultant increased interest in maximizing information from each trials by incorporating biomarker testing (5). While many study designs permit testing of archival tissue, fresh biopsies are often required for reasons such as insufficient quantity/quality as well as possible concerns about heterogeneity and evolution of the marker over time. Prior single institution studies have reported that a biopsy requirement leads to a lengthening of the screening period, however this finding has not been confirmed elsewhere (6).

The impact of a longer screening period for study participation is not well known. Most research protocols do not present the risk associated with a delay in study startup within the informed consent document (7). Cancer research populations consist of people who have progressed on standard of care treatments and are looking for possible salvage therapy, and a delay in time to next treatment may be relevant for this group. Our study aimed to objectively measure how requiring a mandatory research biopsy prior to clinical trial treatment could influence the clinical trial process. We focused our research on the analysis of time from biopsy/trial consent to treatment, time on clinical trial until progression of disease, total time on clinical trial, biopsy complications, and number of major/minor adverse events caused by treatment.

## Materials and Methods

### Study Population and Data Collection

This study was approved by the University of Southern California's Health Sciences Institutional Review Board (HS-18-00169) and included patients located at the USC Norris Comprehensive Cancer Institute in Los Angeles. The study population is one subset of patients seen by research nurses who operated through the standard head/neck and lung team not including patients in the phase 1 program. All of the patients were enrolled in clinical trials from January 16, 2013 to May 28, 2018. Electronic medical records were used to identify such cases.

Information obtained included patient demographics such as the age and type of cancer diagnosis, their race, and their gender. Patients were eligible for this study if they passed their specific clinical trial screening requirements. Written informed consent was obtained by clinical trial personnel from all participants in this study. Four patients were removed from analysis for various reasons including one case with incomplete data in their chart, two subjects disqualified from trial prior to start due to development of study exclusions and disease, randomization to control group which led to withdrawal of consent to participate in study in one subject.

Eligible patients were assigned to one of two groups. One group, the research biopsy group, required the procurement of a fresh tissue biopsy for biomarker testing either prior to clinical trial consent or as part of the study protocol after consent and prior to treatment. The other group, the non-biopsy group, included patients in clinical trials which allowed the use of archival tissue for biomarker testing and did not require a fresh tumor sample prior to treatment. There was also a third group of patients analyzed independently which we labeled as the incidental biopsy group. These were patients who received biopsies to determine recurrence or metastases of their disease. The reason the incidental biopsy group was not included in the analysis is that we were not able to confidently place these patients in either group. They could not be placed in the no biopsy group because they had just undergone a biopsy, and they could not be placed in the research biopsy group because they received the biopsy for reasons other than for the clinical trial itself. Having these patients would affect the analysis by reducing the time until treatment for whichever group they were placed into.

The clinical course of each patient was followed by analysis of outpatient and inpatient hospital records. The following information regarding clinical trial treatment was obtained: number of prior lines of therapy, date of clinical trial consent, date of biopsy consent, biopsy complications, dropout prior to treatment, initiation date of clinical trial treatment, end date of clinical trial treatment, date of progression of disease, and adverse reactions to treatment leading to clinical trial treatment stoppage (classified as major complication) or dose reduction (classified as minor complication).

In order to calculate lines of treatment, we counted each surgical resection, radiation therapy, and chemotherapy regimen as one line of therapy. Multiple surgeries done in one hospitalization were counted as one line of treatment. Maintenance therapy was not counted as an additional line of therapy.

### Data Analysis

Data analysis was performed by calculating the number of days between biopsy consent or clinical trial consent until first treatment, the days between first treatment and progression of disease, and the total days on clinical trial treatment.

The data from the two groups were split, and the median with range or percentage of total were calculated. An unpaired-t test was then applied to applicable data. A p value less than 0.05 was considered statistically significant.

## Results

### Demographic Information

A total of 76 research subjects (64.5% male) were included in this analysis. The highest percentage (39.4%) of the patients were white. This was followed by Hispanic (27.6%) and Asian (21.1%). The remainder were black or other. The highest percentage (56.6%) were adenocarcinomas of the lung. This was followed by 32.9% head/neck cancers. The remainder included mesothelioma, thyroid, and squamous cell carcinoma of the lung (Table 1).

**Table 1:** Demographic information, distribution of different phases and randomizations of clinical trial studies, and reasons for trial stoppage for both groups.

	**Total**	**Research biopsy**	**No biopsy**
**Patients**	76	33 (43.4%)	43 (56.6%)
**Median age at diagnosis**		59 (45–81)	60 (29–85)
**Median number of prior therapies**		2 (0–9)	2 (0–9)
**Median age at initiation of treatment**		62 (48–83)	62 (29–85)
**Sex**			
Male	49 (64.5%)	23 (69.7%)	26 (60.5%)
Female	27 (35.5%)	10 (30.3%)	17 (39.5%)
**Race**			
White	30 (39.4%)	13 (39.4%)	17 (39.5%)
Black	5 (6.6%)	1 (3.0%)	4 (9.3%)
Hispanic	21 (27.6%)	12 (36.4%)	9 (20.9%)
Asian	16 (21.1%)	6 (18.2%)	10 (23.3%)
Other	4 (5.3%)	1 (3.0%)	3 (7.0%)
**Malignancy**			
Mesothelioma	3 (3.9%)	0 (0%)	3 (7.0%)
Thyroid	2 (2.6%)	0 (0%)	2 (4.7%)
Squamous cell carcinoma of the lung	3 (3.9%)	1 (3.0%)	2 (4.7%)
Head/Neck	25 (32.9%)	10 (30.3%)	15 (34.9%)
Adenocarcinoma of the lung	43 (56.6%)	22 (66.7%)	21 (48.8%)
**Clinical trial studies**			
Phase			
I	2 (2.6%)	1 (3.0%)	1 (2.3%)
II	43 (56.6%)	20 (60.6%)	23 (53.5%)
III	6 (7.9%)	3 (9.1%)	3 (7.0%)
IV	0 (0%)	0 (0%)	0 (0%)
I/II	21 (27.6%)	9 (27.3%)	12 (27.9%)
II/III	1 (1.3%)	0 (0%)	1 (2.3%)
III/IV	3 (3.9%)	0 (0%)	3 (7.0%)
Randomization			
Yes	40 (52.6%)	22 (66.7%)	18 (41.9%)
No	36 (47.4%)	11 (33.3%)	25 (58.1%)
**Reasons for trial stoppage**			
Still on trial	8 (10.5%)	3 (9.1%)	5 (11.6%)
Lost to follow up	5 (6.6%)	0 (0%)	5 (11.6%)
Patient decision	2 (2.6%)	1 (3.0%)	1 (2.3%)
Hospitalized	3 (3.9%)	1 (3.0%)	2 (4.7%)
Progression of disease	30 (39.5%)	17 (51.5%)	13 (29.5%)
Death	2 (2.6%)	1 (3.0%)	1 (2.3%)
Major SE	25 (32.9%)	9 (27.3%)	16 (37.2%)
Worse performance status	1 (1.3%)	1 (3.0%)	0 (0%)

### Clinical Trial Details

Overall there were 32 different studies (Supplementary Table 1). The majority of them, 43 (56.6%), were phase II studies. The remainder were phase I to phase IV or a combination of the aforementioned. 40 (52.6%) were randomized trials. There were not significant differences between the two groups in terms of clinical trial phase or randomization (Table 1). None of the clinical trials included in this study required serial biopsies.

### Treatment Data

Out of 76 patients who were analyzed in this study, 56.6% were in the no biopsy group and the remaining 43.4% were in the research biopsy group. The median age of diagnosis was similar for both groups, 60 years old for the no biopsy group and 59 years old for the research biopsy group. The median age at time of trial initiation was equal at 62 years old for both groups.

**Figure 1:**
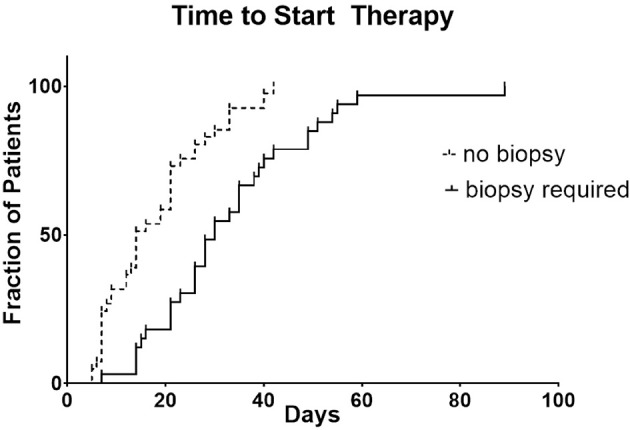
Step-plot comparing days until start of trial for research biopsy and no biopsy groups.

For the research biopsy group, the time from biopsy consent or initial clinical trial consent (whichever occurred first) to clinical trial treatment was 30 days compared to 14 days for no biopsy group (*p* < 0.0001) (Figure 1).

The clinical trial treatment was stopped due to progression of disease either clinically or radiographically in 51.5% of the research biopsy group and 29.5% in the no biopsy group. Major adverse effects due to the treatment 27.3 and 37.2% for research biopsy and no biopsy, respectively. Other reasons for trial stoppage include patient decision to stop, death, hospitalization, and major adverse effects. Some patients are still currently on trial (9.1 vs. 11.6%). For patients who had progression of disease, the median time the research biopsy group was on treatment was for 112 vs. 119 days for no biopsy group (*p* = 0.6605).

**Figure 2:**
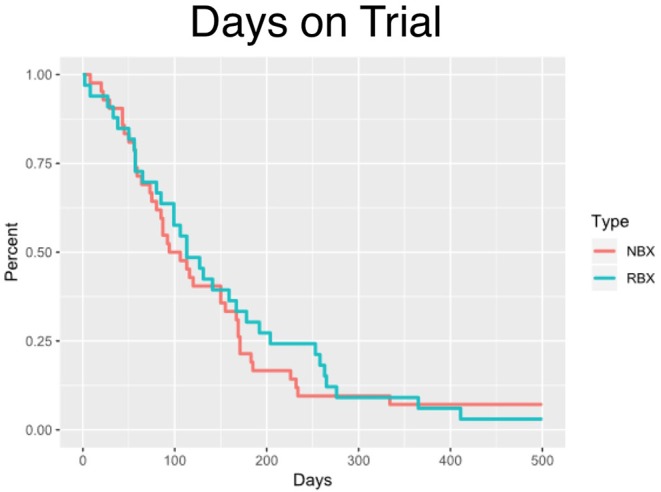
Step-plot comparing days on trial of research biopsy and no biopsy groups.

The median time on clinical trial treatment, which includes those who have progressed and remained on trial, was 112 vs. 105 days (*p* = 0.5732) for research biopsy vs. non-biopsy groups, respectively (Figure 2).

In the research biopsy group, 7 (21.2%) of the patients received clinical biopsies prior to clinical trial consent which served as the required research biopsy. The other 26 (78.8%) had their research biopsies taken after the clinical trial consent was signed. 3 (9.1%) of all patients who received research biopsies had complications due to biopsy. These complications included: pneumothorax after CT-guided lung biopsy which resulted in an overnight hospital stay, trace pneumothorax after CT-guided lung biopsy, and intractable pain after liver biopsy which resulted in refusal of future procedures. No patients in the research biopsy group dropped out due to complications from the biopsy itself. When comparing major adverse effects, to treatment (16 days, *p* < 0.0001).

This corroborates the findings in a study done by Spiegel et al. comparing similar two groups which showed a statistically significant 20 day delay in the start of treatment (6). These two studies show that mandatory research biopsies for clinical trial enrollment affect which we defined as an adverse effect from the treatment which required stoppage of trial treatment, the research biopsy group had 9 (27.3%) vs. the no biopsy group with 16 (37.2%) (*p* = 0.36.38). For minor adverse effects, defined as adverse effects from the treatment resulting in dose reduction, the research biopsy group had 7 (21.2%) vs. the no biopsy group with 5 (11.6%). All data mentioned above can be found in Tables 1, 2.

**Table 2:** Complete chart comparing the research biopsy and no biopsy group.

	**Research biopsy**	**No biopsy**	**Unpaired *t*-test**
Total Number	33 (43.4%)	43 (56.6%)	
	**Median**	**Median**	
Number who received research biopsy prior to consent	7 (21.2%)	0	
Number who received research biopsy after consent	26 (78.8)	0	
Number of Research Biopsy Complications	3 (9.1%)	0	
Time from research biopsy to initial consent (d)	2 (−20 to 44)	n/a	
Time from initial consent to research biopsy (d)	15.5 (0–42)	n/a	
Number of dropout	0 (0%)	0 (0%)	
Time from research biopsy to clinical trial treatment (d)	14.5 (5–89)	n/a	
Time from initial consent to clinical trial treatment (d)	28 (5–59)	14 (0–42)	
Time from research biopsy/clinical trial consent to treatment (longer duration used) (d)	30 (7–89)	14 (0–42)	*p* < 0.0001
Time on trial before disease progression (d)	112 (49–410)	119 (42–957)	*p =* 0.6605
Total time on clinical trial (d)	112 (1–509)	105 (7–1360)	*p =* 0.5732
Number of major adverse effect	9 (27.3%)	16 (37.2%)	*p =* 0.3638
Number of minor adverse effect	7 (21.2%)	5 (11.6%)	*p =* 0.2582

## Discussion

The analysis of this study showed that clinical trials that included mandatory research biopsies with biomarker testing resulted in a statistically significant delay in the start of treatment when compared to clinical trials which did not require biopsies prior treatment start dates. This may be a topic of interest when making future guidelines regarding mandatory tissue samples for clinical trials.

Potential factors that could also contribute to this delay include requirements for washout of previous chemotherapy treatments which on average for both groups was approximately 28 days as well as difficulty scheduling patients for imaging and clinic visits.

Other data in this study such as the difference in time on clinical trial until progression, total time on clinical trial, and percentage of major/minor adverse events due to treatment were not statistically when comparing the two groups. The research biopsy group did have members experience complications due to the biopsy.

There are alternatives such as the use of non-invasive liquid biopsies which may be used in the future which could shorten or eliminate alternatives for future clinical trials. Non-invasive liquid biopsies include the use of body fluids such as blood, saliva, and urine, in order to test for tumor- specific cell-free DNA (cfDNA) or circulating tumor DNA (ctDNA) (8, 9). These tests would be able to be done when a patient initially starts their trial. Another benefit of liquid biopsies would be more frequent monitoring throughout the treatment. However, more studies need to be done to determine their accuracy in monitoring dynamic biomarkers (e.g., met expression).

Clinical trial protocols should prospectively take into account the type of biomarker they are monitoring and if it is essential prior to initiation of treatment. It is important to know whether a biomarker is dynamic vs. non-dynamic as well as determine if certain biomarkers are spatially dynamic. This first point can be done by comparing the expression of biomarkers in fresh vs. archival tissue in order to determine whether there is a change in expression of a specific biomarker, and in essence, determine whether the fresh biopsy is truly necessary and beneficial. Efforts to understand the dynamic nature of biomarkers before designing the biomarker plan in clinical trials should be accomplished early in the course of drug development. Spatially dynamic biomarkers by contrast may be better assessed with tools like CTCs and ctDNA that provide some aggregation of signals across the various anatomic sites.

While the informed consent form the patient has to sign prior to screening is comprehensive in detailing the risks/benefits of the trial process, all of the trials reviewed in this study that required fresh research biopsies did not clearly mention the added delay in treatment when getting this biopsy. Recent ASCO guidelines are silent on the time delay associated with biopsy requirements, and future revisions of those guidelines should incorporate treatment delay as a risk (10). While we did not identify a survival difference between the biopsy and non-biopsy groups in this trial, the study was neither designed or powered to identify survival differences. We can only conclude that a biopsy requirement results in a treatment delay, understanding the impact of that delay on response and survival requires further investigation.

The limitations in this study include use of a single institution, the heterogeneity of malignancies, the multiple types of clinical trial treatments, the retrospective and non-randomized nature of the analysis. Because any prospective study attempting to answer the question of biopsy delays would be impacted by the Hawthorne effect, the real world evidence approach taken here may be the preferred methodology to quantify risk of treatment delay (11).

## Conclusion

While biopsies with subsequent biomarker identification can help individualize therapy for patients, our study shows that requiring biopsies prior to clinical trial treatment results in a statistically significant delay in treatment. The informed consent forms that were part of clinical trials involving mandatory research biopsies did not reflect this delay in treatment. While these delays, did not result in a significantly significant decrease in number of days on trial or days until progression of disease, for those individuals in whom expedient start of therapy is necessary, research biopsy requiring trials may interfere with prompt treatment, both patients and clinicians should understand that risk in the context of the practices within their own health systems.

## Data Availability Statement

The raw data supporting the conclusions of this manuscript will be made available by the authors, without undue reservation, to any qualified researcher.

## Ethics Statement

Authors are required to state the ethical considerations of their study in the manuscript, including for cases where the study was exempt from ethical approval procedures. This research was approved by the University of Southern California Health Science Institutional Review Board HS-18-00169.

## Author Contributions

JC: primary author, submitted IRB proposal, performed data collection and data analysis, created tables and figures, and wrote majority of manuscript. JN: principle investigator, helped with IRB proposal, provided patients included in study, created figure, and reviewed and edited manuscript. JT: performed data collection, helped create tables, wrote, and edited parts of manuscript. AE-K: provided patients included in study and reviewed manuscript. SZ: helped with data collection, figure creation, and manuscript review and editing.

### Conflict of Interest

The authors declare that the research was conducted in the absence of any commercial or financial relationships that could be construed as a potential conflict of interest.
